# Efficacy of tracheostomy for respiratory management in patients with advanced oral cancer

**DOI:** 10.1186/s40902-024-00439-4

**Published:** 2024-07-22

**Authors:** Yun-Ho Kim, Jae-Young Yang, Yoon-Hee Ma, Jin-Choon Lee, Dae-Seok Hwang, Mi-Heon Ryu, Uk-Kyu Kim

**Affiliations:** 1 Department of Oral and Maxillofacial Surgery, School of Dentistry, Pusan National University, Yangsan, Republic of Korea; 2 Department of Otorhinolaryngology-Head and Neck Surgery, School of Medicine, Pusan National University, Yangsan, Republic of Korea; 3 Department. of Oral Pathology, School of Dentistry, Pusan National University, Yangsan, Republic of Korea

**Keywords:** Oral cancer, Tracheostomy, Airway obstruction

## Abstract

**Background:**

Many studies have been reported on tracheostomy to prevent upper airway obstruction after surgery. Among these, the scoring system proposed by Cameron et al. quantifies various factors that influence postoperative respiratory failure. This system provides a basis for surgeons to decide whether to perform an elective tracheostomy. In this study, the authors applied the Cameron scoring system retrospectively to patients undergoing severe oral cancer surgery to reevaluate the indications for elective tracheostomy and to investigate its clinical efficacy in airway management. In this study, a sample of 20 patients who underwent oral cancer surgery was selected and divided into two groups: 10 underwent tracheostomy and 10 did not. The Cameron scoring scores for each patient were extracted, to verify whether elective tracheostomy was performed in accordance with the threshold scores. Differences in scores and significant clinical impact factors between the two groups were analyzed and compared.

**Result:**

The 10 patients who underwent tracheostomy had an average Cameron score of 6.4, all scoring above the recommended threshold of 5 for tracheostomy. For the 10 patients who did not undergo tracheostomy, the average score was 2.5, with 8 out of these 10 patients scoring below 5. Significant clinical impact factors observed included the location and size of the tumor, the performance of mandibulectomy and neck dissection, and the type of reconstruction surgery.

**Conclusion:**

In planning surgery for oral cancer patients, it is essential to consider the use of elective tracheostomy based on preoperative assessment of the risk of postoperative airway obstruction using tools like the Cameron scoring system, and patients’ condition. Research confirms that elective tracheostomy effectively enhances airway management in patients with severe oral cancer.

## Background

Severe oral cancer surgery carries the risk of inducing upper airway obstruction after the procedure. Particularly, resections of the mandible, tongue, or floor of the mouth can lead to delayed bleeding and subsequent swelling of the posterior regions of the tongue and the pharynx, significantly increasing the risk of upper airway obstruction. The need for tracheostomy in head and neck surgeries to prevent this has been well-established by various previous studies, yet whether to perform elective tracheostomy varies depending on various factors specific to each surgical case. Clinically, these factors often include the degree of postoperative neck swelling and also the surgeon’s ability to predict the volume of the reconstruction flap. Although reconstructive surgery using free flap techniques offers many benefits, complications can still occur postoperatively from free flap reconstructions.

The indications for tracheostomy are manifold, but its primary purpose is to ensure the safe control of the upper airway and improve respiration by securing the airway. Tracheostomy facilitates safe airway management and is widely used by many surgeons. However, the optimal method of airway management after cancer surgery remains controversial, and tracheostomy does not always guarantee success in airway management. Performing a tracheostomy can interfere with the patient’s laryngeal function, impede proper humidification, and often cause complications such as coughing, excessive secretion, and pulmonary issues [1]. Other complications of tracheostomy include infection, bleeding, tracheomalacia, tracheoesophageal fistula (TEF), tracheo-innominate artery fistula, tube obstruction, and so on [2–4]. Therefore, it is not always necessary to perform tracheostomy in head and neck cancer surgeries, and its use should be selectively considered only when absolutely necessary. However, determining when it is absolutely necessary often relies on the subjective judgment of the surgeon, and several clinical studies have been conducted to provide a more objective basis for decision-making.

Most notably, a scoring system that quantifies various factors to be considered when performing a tracheostomy was proposed by Cameron [5]. The Cameron scoring system evaluates factors influencing the decision to perform elective tracheostomy in head and neck malignancy surgeries. This scoring system consists of four main categories: the site of the tumor, whether a mandibulectomy was performed, whether bilateral neck dissection was conducted, and the type of flap used in the reconstruction. Points are assigned within each category based on the expected clinical impact on neck swelling and airway obstruction. It is suggested that if the combined score from these four categories exceeds a threshold of 5 points, there is a high risk of upper airway obstruction, and an elective tracheostomy should be considered.

In this study, oral cancer surgery patients at the Department of Oral and Maxillofacial Surgery, Pusan National University Dental Hospital, were divided into groups of those who had undergone tracheostomy and those who had not. The Cameron scoring system was retrospectively applied to assess its clinical usefulness. Additionally, the study aimed to present considerations for deciding whether to perform a tracheostomy, by comparing other various scoring systems related to the execution of tracheostomy during oral cancer resection surgeries.

## Materials and methods

During surgery for oral cancer patients, the appropriateness of elective tracheostomy was evaluated using the Cameron scoring system. The study examined 20 oral cancer surgery patients classified as severe cases, who underwent surgery at the Department of Oral and Maxillofacial Surgery, Pusan National University Dental Hospital from 2010 to 2021. The cases were selected based on indications for surgical resection and reconstruction, and the investigation included variables such as the primary tumor location and size, type of resection, type of flap used for reconstruction, type and location of neck dissection, duration of stay in the intensive care unit (ICU) and ward, ASA(American Society of Anesthesiologists) grade, total operation time, duration of keeping tracheostomy or nasotracheal tube, and smoking status.

The patients were divided into two groups: 10 who underwent tracheostomy and 10 who did not. Scores from the Cameron scoring system were compared to determine if tracheostomy was performed in cases where the score was 5 or above, and whether there were cases with scores above 5 that did not receive a tracheostomy. Conversely, we sought to detect whether there were cases with scores below 5 where a tracheostomy was performed. This analysis was conducted to assess the usefulness of the Cameron scoring system and the efficacy of tracheostomy in actual clinical application.

The score calculation was based on the scoring system provided by Cameron [5] in the literature (Table 1). Additionally, for comparison and analysis with other scoring systems, the scoring systems proposed by Mohamedbhai et al. (TRACHY system) [6] and Gupta et al. (CASST system) [7] are also described.

**Table 1:** Tracheostomy scoring systems by Cameron (2009), Mohamedbhai (2016), and Gupta (2016)

Cameron et al. (2009)		Mohamedbhai et al. (2016) (TRACHY)		Gupta et al. (2016) (CASST)	
Variables	Score	Variables	Score	Variables	Score
**Tumor site**		**T stage**		**Major criterion**	
Cutaneous	0	T1–T2	0	Previous RT head and neck	2
Oral cavity		T3–T4	1		
Buccal mucosa	0	**Reconstruction**		Two or more subsites of the oral cavity or oropharynx	2
Maxilla	0	Fasciocutaneous	0		
Mandibular alveolus	1	Myocutaneous or composite	1		
Anterior tongue	1	Two flaps	3	Bilateral neck dissection	2
Floor of mouth	2	**Anatomy**		Bulky flap*	2
Oropharynx		Lateral or central	0	Extended hemi or central arch mandibulectomy	2
Soft palate	3	Anterior or oropharyngeal	2		
Anterior pillar	3				
Tonsillar pillar	4	**Coexisting conditions**		Flap with a compressing element (reconstruction plate)	2
Posterior tongue	4	ASA 1 or ASA 2	0		
Hypopharynx	4	ASA 3	1		
**Mandibulectomy**		**History**		**Minor criterion**	
No	0	None	0	Age ≥ 65 years	1
Yes	1	Previous operation head and neck	1	Previous operation on the same site	1
**Bilateral neck dissection**		Previous RT head and neck	3		
No	0			Pathological CT chest findings (COPD, etc.)	1
Yes	3	**Neck dissection**			
**Reconstruction**		None	0		
None	0	Unilateral	0	Trismus (< 1 cm)	1
RFFF	2	Bilateral	3		
Other	3				
**Tracheostomy recommended score**	≥ 5		≥ 4		≥ 7

*RFFF* radial forearm free flap, *RT* radiation therapy

^*^Latissimus dorsi, double skin island pectoralis major myocutaneous flap

Patients undergoing elective tracheostomy typically had the procedure to be planned and performed in advance of surgery. However, the study also included cases where emergency tracheostomy was necessary, such as in unforeseen postoperative respiratory failures. These particular cases were analyzed separately later. The average scores for each group were examined to determine whether the scores for individual patients matched the actual performance of tracheostomy, or if there were discrepancies. Statistical analysis was performed using IBM SPSS Statistics 29.0.2.0 (SPSS Inc., Chicago, IL) software, with a significance level of 0.05 or less. Due to the relatively small sample size, the correlation between each factor investigated and the Cameron score was compared using Spearman’s rank correlation analysis, and differences between the two groups were compared using the Mann–Whitney *U* test. For the correlation between the surgical site and the Cameron score, the Kruskal–Wallis test was used to analyze categorical variables with three or more groups.

## Results

In this study, the 10 patients who underwent tracheostomy had an average Cameron score of 6.4, all of whom scored above the threshold of 5 points. For the 10 patients who did not undergo tracheostomy, the average score was 2.5, with 8 out of these 10 patients scoring below the threshold of 5 points, demonstrating a general consistency between Cameron’s recommended threshold score of 5 for elective tracheostomy and the actual performance of tracheostomy. One patient, not initially planned for tracheostomy before surgery, underwent emergency tracheostomy on the eighth day post-surgery due to respiratory suppression caused by swelling (patient 7 in the tracheostomy group). This patient received a Cameron score of 6 points retrospectively, positioned slightly more towards the high-risk group on the borderline between high and low risk. The Cameron scores for each patient, based on the surgical resection type and site, neck dissection, and reconstruction, were recorded separately in Table 2 (tracheostomy group) and Table 3 (non-tracheostomy group).

**Table 2:** Cameron score of 10 patients who underwent tracheostomy

No	Age	Sex	Excision	Neck dissection	Flap	Site	Cameron Score
1	60	M	Marginal mandibulectomy (1)	SOHND (Rt.), SND (Lt.) (3)	RFFF (2)	Rt. FOM (2)	8
2	55	M	Segmental mandibulectomy (1)	SOHND(both) (3)	FFF (3)	both FOM (2)	9
3	54	M	Segmental mandibulectomy (1)	SOHND(both) (3)	FFF (3)	Mn. ant. (1)	8
4	60	M	Wide excision	SOHND(Lt.)	RFFF (2)	Tonsil, Retromolar area (4)	6
5	39	M	Segmental mandibulectomy (1)	SOHND(Rt.)	FFF (3)	Mn. Rt. (1)	5
6	61	F	Marginal mandibulectomy, partial glossectomy (1)	SOHND(Lt.)	RFFF (2)	Tongue base, Mn. Lt. (4)	7
7	71	M	Partial glossectomy	SOHND(Lt.)	RFFF (2)	Central part of tongue (4)	6
8	52	F	Segmental mandibulectomy (1)	SOHND(Rt.)	FFF (3)	Mn. Rt. (1)	5
9	70	M	Segmental mandibulectomy (1)	SOHND(Lt.)	FFF (3)	Mn. Lt. (1)	5
10	66	F	Segmental mandibulectomy (1)	SOHND(Lt.)	FFF (3)	Mn. Rt. (1)	5
Mean	58.8						6.4

*(  )* each item’s Cameron score, *SOHND* supraomohyoid neck dissection, *SND* selective neck dissection, *FOM* floor of mouth, *FFF* fibula free flap

**Table 3:** Cameron score of 10 patients who did not undergo tracheostomy

No	Age	Sex	Excision	Neck dissection	Flap	Site	Cameron score
1	52	M	Hemiglossectomy	mRND(Rt.)	RFFF (2)	Rt. lat. border of tongue (1)	3
2	81	M	Marginal mandibulectomy (1)	SOHND(Lt.)	RFFF (2)	Mn. Lt. (1)	4
3	69	M	Segmental mandibulectomy (1)	SOHND(Lt.)	FFF (3)	Mn. Lt. (1)	5
4	49	M	Segmental mandibulectomy (1)	SOHND(Lt.)	FFF (3)	Mn. Lt. (1)	5
5	57	M	Partial maxillectomy	SOHND(Rt.)	Buccal fat pad	Rt. Mx. Post	0
6	70	M	Partial maxillectomy	None	None	Lt. Mx. Post	0
7	62	M	Subtotal maxillectomy	SND(Rt.)	None	Rt. Mx. Ant. and Post	0
8	68	F	Wide excision	SOHND(Lt.)	RFFF (2)	Lt. buccal mucosa	2
9	77	F	Wide excision	SOHND(Rt.)	RFFF (2)	Mn. Rt. (1)	3
10	69	F	Wide excision	SOHND(Lt.)	RFFF (2)	Mn. Lt. (1)	3
Mean	65.4						2.5

*(  )* each item’s Cameron score. *mRND*, modified radical neck dissection

In the results of Tables 2 and 3, the Cameron scores of the two groups were tested and compared using Mann–Whitney analysis, revealing a significant score difference between the two groups with *p* < 0.001. Additionally, factors that could affect airway management, and factors required for scoring in other systems (TRACHY, CASST), such as age, sex, T stage of the tumor, bilateral neck dissection, length of stay in ICU (intensive care unit) and ward, ASA grade, smoking, duration of tracheostomy or nasotracheal tube keeping, operation time, use of metal plates, and whether it was a reoperation, are investigated and summarized in Tables 4 and 5, respectively. There was no patient with a history of prior radiation therapy to the surgical site.

**Table 4:** Clinical factors and scores of 3 tracheostomy scoring systems of 10 patients who underwent tracheostomy

No	Age	Sex	Stage	bND	ICU days	Hospital days	ASA grade	Smoking	Days with tracheostomy tube keeping	Operation time (min)	Use of Recon. plate	Re-OP.	Cameron (threshold: 5)	TRACHY (threshold: 4)	CASST (threshold: 7)
1	60	M	T3	O	0	44	1	-	20	855	-	-	8	6	2
2	55	M	T4	O	0	59	1	-	15	900	O	-	9	7	6
3	54	M	T3	O	1	104	2	O	41	990	O	O	8	8	7
4	60	M	T2	-	1	38	1	O	13	630	-	-	6	2	0
5	39	M	T4	-	4	34	1	O	7	645	O	-	5	2	2
6	61	F	T4	-	4	39	1	O	20	960	-	-	7	3	2
7	71	M	T2	-	5	45	2	-	27	765	-	-	6	0	1
8	52	F	T4	-	2	85	1	-	18	750	O	-	5	4	4
9	70	M	T3	-	3	187	2	O	11	825	O	-	5	2	3
10	66	F	T4	-	3	58	1	-	8	730	O	O	5	3	4
Mean	58.8				2.3	69.3			18	805			6.4 (10/10)	3.7 (4/10)	3.1 (1/10)

*bND* bilateral neck dissection, *ICU* intensive care unit, *Recon.* reconstruction, *Re-OP.* re-operation on the same site

No patients received previous radiation therapy on operation site

**Table 5:** Clinical factors and scores of 3 tracheostomy scoring systems of 10 patients who did not underwent tracheostomy

No	Age	Sex	Stage	bND	ICU days	Hospital days	ASA grade	Smoking	Days with nasotracheal tube keeping	Operation time (min)	Use of Recon. plate	Re-OP.	Cameron (threshold: 5)	TRACHY (threshold: 4)	CASST (threshold: 7)
1	52	M	T2	-	5	36	1	-	0	145	-	O	3	1	1
2	81	M	T2	-	7	39	2	-	5	800	O	-	4	0	3
3	69	M	T4	-	0	59	2	-	4	900	O	-	5	2	3
4	49	M	T4	-	0	64	2	-	4	810	O	O	5	3	3
5	57	M	T2	-	0	35	2	-	1	360	-	-	0	0	0
6	70	M	T2	-	0	42	2	-	1	270	-	-	0	0	1
7	62	M	T2	-	0	51	2	-	0	510	-	-	0	0	0
8	68	F	T2	-	0	40	1	-	1	585	-	-	2	0	1
9	77	F	T2	-	0	65	2	-	1	630	-	-	3	0	1
10	69	F	T4	-	0	73	2	-	2	600	-	-	3	1	1
Mean	65.4				1.2	50.4			1.9	561			2.5 (2/10)	0.7 (0/10)	1.4 (0/10)

No patients received previous radiation therapy on operation site

The average age of patients in the group that underwent tracheostomy was 58.8 years, compared to 65.4 years for those who did not undergo tracheostomy. Regarding the duration of stay in ICU, the average stay was 2.3 days for the tracheostomy group and 1.2 days for the non-tracheostomy group. The average length of stay in the ward was 69.3 days for the tracheostomy group and 50.4 days for the non-tracheostomy group.

There were a total of 5 smokers in the tracheostomy group, specifically patients number 3, 4, 5, 6, and 9, while all 10 patients in the non-tracheostomy group were non-smokers. All patients were classified as ASA grade I or II, meaning they were either healthy or had only mild systemic disease that did not pose significant issues for general anesthesia surgery. The average duration of tracheal tube maintenance in the tracheostomy group was 18.0 days, and the average duration of nasotracheal intubation in the non-tracheostomy group was 1.9 days. The average operation time was 805 min in the tracheostomy group and 561 min in the non-tracheostomy group. It was the only clinical factor that showed a statistically significant difference between the two groups (*p* = 0.015), aside from the Cameron score (*p* = 0.001). Furthermore, in the non-tracheostomy group, longer operation times were associated with longer postoperative nasotracheal tube maintenance durations (*p* = 0.004).

Additionally, to assess the appropriateness of the Cameron system compared to other scoring systems, the TRACHY and CASST systems were applied to these cases. The results showed that, among the 10 patients who underwent tracheostomy, all 10 met the tracheostomy criteria according to the Cameron system, whereas 4 out of 10 met the criteria according to the TRACHY system, and only 1 out of 10 met the criteria according to the CASST system.

Finally, the correlation between each investigated item and the Cameron score was analyzed and summarized in Table 6. A higher tumor stage and bilateral neck dissection were correlated with higher Cameron scores. There was also a significant correlation based on the surgical site. In post hoc testing after the Kruskal–Wallis test, which analyzes the influence of variables in three or more groups, a significant difference was found between the mandibular alveolus and anterior tongue areas (assigned 1 point in the Cameron score) and the floor of the mouth area (assigned 2 points in the Cameron score). Additionally, higher Cameron scores tended to correlate with longer durations of tracheostomy or nasotracheal intubation, and also longer operation times. There was no significant correlation between the Cameron scores and factors such as age, sex, length of hospital stay, overall health, or smoking status.

**Table 6:** Correlation of each factor with the Cameron score

Factors correlated with the Cameron score	Age	Sex**	Stage	Site***	bilateral neck dissection	ICU days	Hospital days	ASA grade	Smoking	Days with tracheostomy tube keeping	Days with nasotracheal tube keeping	Operation time (min)	Use of Recon. plate	Re-OP.
*P*-value	0.233	0.241	0.03*	0.002*	0.003*	0.276	0.372	0.106	0.053	0.049*	0.018*	0.001*	0.106	0.610

Statistical analysis by Spearman correlation analysis

^*^p-value < 0.05

^**^Statistical analysis by Mann–Whitney test

^***^Statistical analysis by Kruskal–Wallis test

## Discussion

After oral cancer surgery, there are various airway management strategies such as immediate extubation, delayed extubation after maintaining intubation for a specific period, or tracheostomy. Consequently, there is a diversity of research concerning postoperative airway management in patients with oral cancer. Regarding tracheostomy, numerous studies suggest that tracheostomy is not always necessary in most cases. Additionally, researches on scoring systems designed to objectively assess the need for tracheostomy before surgery have reported on factors that increase the necessity for performing a tracheostomy.

In 2005, Mishra et al. [8] conducted a retrospective study on airway management in oral cancer surgery patients and mentioned that maintaining nasal intubation without tracheostomy could be a sufficiently safe alternative. Moreover, in 2022, Madgar et al. [9] investigated the necessity of tracheostomy after microvascular reconstruction in head and neck surgeries, stating that elective tracheostomy should be considered on case-by-case basis and that its routine use is unnecessary. Additionally, their study reported that patients who did not receive elective tracheostomy were younger and had shorter postoperative hospital stays. Similar results were confirmed in this study. In this study, the duration of ICU and ward stays for each patient was investigated, showing an average of 2.3 days and 69.3 days, respectively, in the group that underwent tracheostomy and 1.2 days and 50.4 days in the group that did not, suggesting a trend of reduced duration of hospitalization when tracheostomy was not performed. However, the average age of patients was 58.8 years in the tracheostomy group and 65.4 years in the non-tracheostomy group, showing a higher average age in the group that did not receive tracheostomy. Although no direct causal relationship between age and the tendency to perform tracheostomy was observed, it was noted in this study that the correlation between patient age and tracheostomy is not significant. The decision to perform tracheostomy was primarily based on the extent of the resection and the location of the primary lesion.

Another study by Myatra et al. [10] suggested that a delayed extubation strategy after oral cancer surgery could be a method with fewer complications and beneficial for reducing hospital stays and aiding recovery, compared to tracheostomy. This study identified independent predictors for safely choosing delayed extubation, including smaller primary tumors of stages T1–T2, absence of extensive resection, primary closure or reconstruction using a fasciocutaneous flap, no preoperative radiotherapy, no neck dissection or unilateral neck dissection only, and shorter anesthesia duration. Currently, our institution primarily uses nasal intubation for immediate post-surgery airway management according to these criteria. We employ the Cameron scoring system preoperatively to assess the risk of respiratory failure and decide on the necessity of elective or emergency tracheostomy postoperatively when necessary.

According to the Cameron scoring system referenced in this study, patients with scores of 5 or above are classified as high risk, while those with scores below 5 are considered low risk. Cameron reported that tracheostomy might be considered for patients classified as high risk. In this study, the group that underwent tracheostomy had an average Cameron score of 6.4, with all 10 patients scoring 5 or more. In the group that did not undergo tracheostomy, discrepancies between the Cameron score and the actual performance of tracheostomy were observed. The average Cameron score in this non-tracheostomy group was 2.5, and although 8 out of 10 patients scored below the threshold, 2 patients (patients number 3 and 4 in the non-tracheostomy group) scored the baseline 5 points for preoperative tracheostomy consideration but maintained their airway with only nasal intubation without significant complications.

However, maintaining the airway via nasal intubation can lead to worsening bronchial swelling, sputum deposition, and throat pain if it extends beyond 3 days. Therefore, it was thought that elective tracheostomy might be necessary for cancer surgeries requiring more than 5 days of artificial airway maintenance. In this study, during respiratory management by nasotracheal tube, the tube was typically removed within 3 days, with an average extubation time of 1.9 days. For tracheal tube respiratory management, the tube was removed on the 5th postoperative day in early cases, or a new tube was exchanged once based on the healing status of the surgical site, and then completely removed between the 10th and 14th postoperative days. In this study, tracheostomy and tube removal were performed by ENT doctors, and the tracheostomy tube removal was performed on average 18.0 days postoperatively. Both nasotracheal tube and tracheal tube cases managed their airways well without any serious complication. Patient 2 in the non-tracheostomy group (Tables 3 and 5), who maintained non-tracheal intubation for more than 5 days, received mechanical ventilation management in the ICU before being transferred to the ward. However, patient 7 in the tracheostomy group (Tables 2 and 4), who was kept in the nasotracheal intubation postoperatively, complained of airway discomfort and dyspnea during mechanical ventilation management, leading to a late tracheostomy. After being transferred to the ICU, the patient’s respiratory discomfort was resolved with 3 days of intensive mechanical ventilation treatment, allowing them to return to the ward.

An interesting point from the results is that higher Cameron scores not only correlated with longer tracheal tube maintenance periods in the tracheostomy group but also significantly increased intubation durations in the naturally intubated (non-tracheostomy) patient group. Therefore, this observation is significant as it suggests that preoperative evaluation using the Cameron score can aid in predicting and planning postoperative airway management, not only for patients undergoing tracheostomy but also for those not undergoing the procedure.

Although the Cameron scoring system’s threshold score generally matched the performance of tracheostomy in this study, there were instances in actual clinical practice where tracheostomy was performed or deemed unnecessary, contrary to predictions made using the scoring system. A similar retrospective analysis by Lee et al. [11] reported that among 22 patients with a Cameron score of 5 or higher, only 5 (22.7%) underwent tracheostomy. Additionally, tracheostomies are typically performed electively before cancer ablation surgeries. However, if prolonged mechanical ventilation is anticipated postoperatively, clinicians often consider switching from nasal intubation to a tracheostomy tube. Although there is no set optimal timing, it is common for clinicians to perform a tracheostomy if the expected maintenance period of nasal intubation exceeds 5 days, due to patient discomfort and the risk of pneumonia.

Patient number 7 from the tracheostomy group in this study, who was not initially planned for a tracheostomy, underwent emergency tracheostomy on the eighth day post-surgery due to respiratory suppression caused by tongue swelling. This patient had a Cameron score of 6 points, placed slightly more in the high-risk category than the borderline between high and low risk. Fortunately, after the tracheostomy, the patient’s respiratory failure resolved, and the surgical site healed properly, allowing for a normal discharge. This case could have been considered more actively for elective tracheostomy before surgery if they had been identified as high risk through objective indicators like the Cameron scoring system beforehand. This case exemplifies the usefulness of the Cameron scoring system. Currently, our hospital applies the scoring system before surgery to assess the necessity for tracheostomy.

In this study, none of the patients undergoing maxillectomy received a tracheostomy, and they were actually classified as low risk with Cameron scores below 5. The Cameron scoring system assigns a risk score of 1 point for mandibulectomy, whereas maxillectomy receives 0 points, indicating no increased risk. This is likely because the maxilla is relatively less adjacent and related to the upper airway and areas around the mandible, such as the tongue and floor of the mouth, thus considered to have a lower risk of postoperative airway obstruction.

A study by Lin et al. [12] evaluating the necessity of airway management and tracheostomy after maxillectomy concluded that routine tracheostomy is unnecessary for patients undergoing maxillectomy. In the study, only 10 out of 130 maxillectomy cases (7.7%) underwent tracheostomy, including three cases where mandibulectomy was also performed and four cases involving extensive flap reconstruction. And, some tracheostomies were performed at the surgeon’s discretion due to concerns about upper airway swelling, confirming a very low incidence of tracheostomy with typical maxillectomy surgeries. However, the study also indicates that while the numbers are low, tracheostomies have been performed in some maxillectomy cases, suggesting that tracheostomy should not be categorically ruled out in maxillary surgeries and should be decided based on a thorough preoperative evaluation. In this study, all three cases of maxillectomy maintained airway control via nasal intubation, without the need for tracheostomy.

Some studies suggest that relying solely on a scoring system to decide on tracheostomy can lead to its unnecessary overuse. According to research by Schmutz et al. [13], tracheostomies are widely used to prevent airway obstruction despite various negative impacts on complications and quality of life, and general clinical scoring systems do not prevent tracheostomies but can lead to more unnecessary procedures. They proposed that delayed extubation in the ICU can be a safe and feasible approach that also reduces the rate of tracheostomies. Additionally, a study by Lee et al. [11] mentioned that using Cameron’s scoring system to perform elective tracheostomies could lead to more patients unnecessarily receiving tracheostomies. Indeed, in this study, among the 10 patients who did not undergo tracheostomy, two (patients number 3 and 4) had scores above the threshold of 5, but considering the location of their lesions and overall health condition, airway maintenance was successfully managed with nasal intubation alone, without significant complications. In the other group, all 10 patients who received tracheostomies also recovered well without any bronchial complications.

In a study by Leiser [14], smoking status was also mentioned as a consideration when deciding tracheostomy. This study examined smoking status and found that among the 10 patients who underwent tracheostomy, 5 (patients number 3, 4, 5, 6, and 9) were smokers, while none of the 10 patients in the non-tracheostomy group were smokers. While it is not clear if smoking itself increases the likelihood of needing a tracheostomy, it can be inferred that the necessity might have increased due to larger and more advanced primary lesions associated with smoking.

**Fig. 1:**
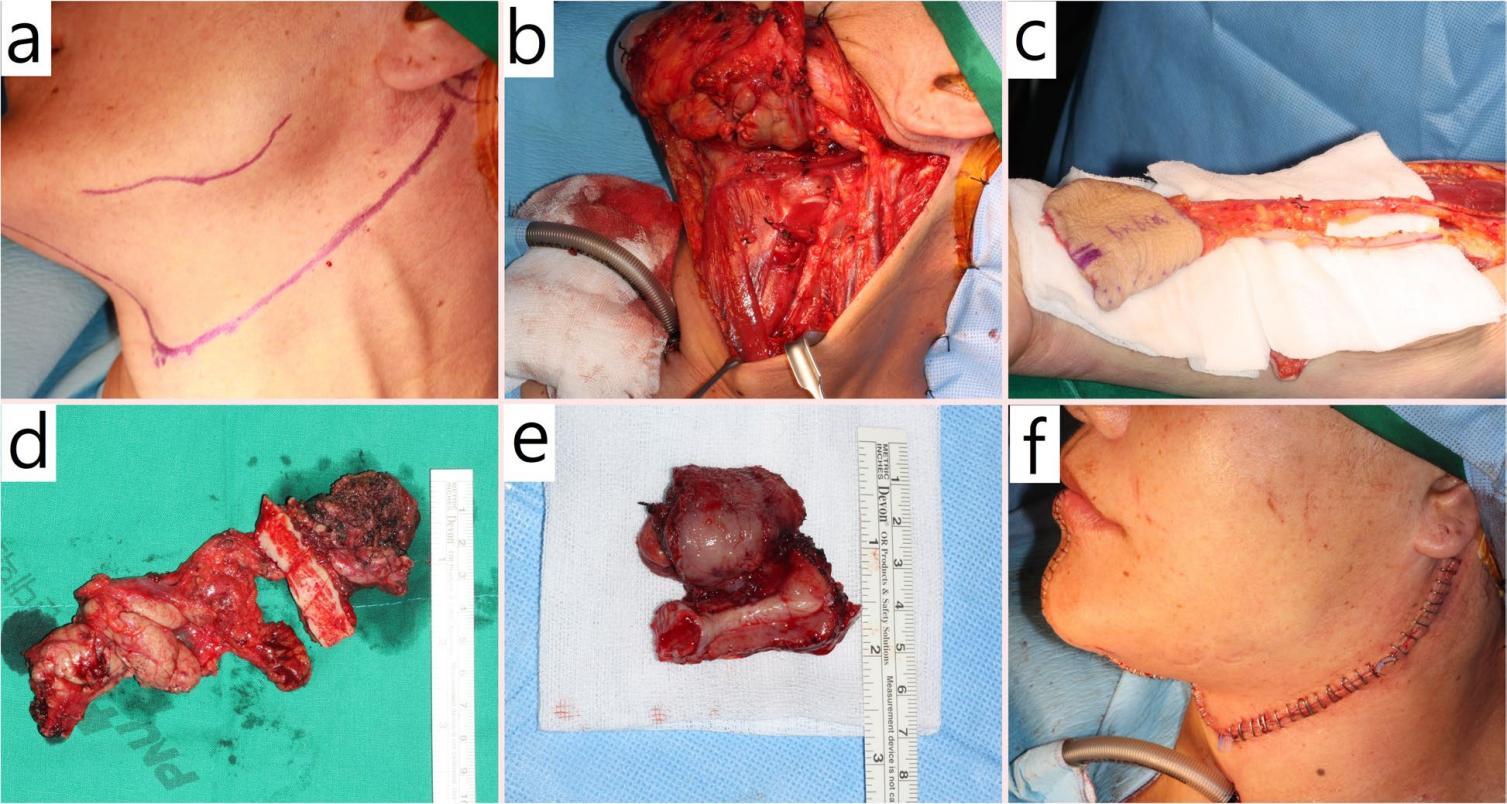
Intra-operation photo of patient No. 6 of group with tracheostomy. **a** Neck incision line drawing state. **b** Neck dissection with tracheostomy. **c** Radial forearm flap harvesting. d Excised lymphatic chains. **e** Excised tongue mass and adjacent tissues. **f** Primary closure with tracheostomy James tube

**Fig. 2:**
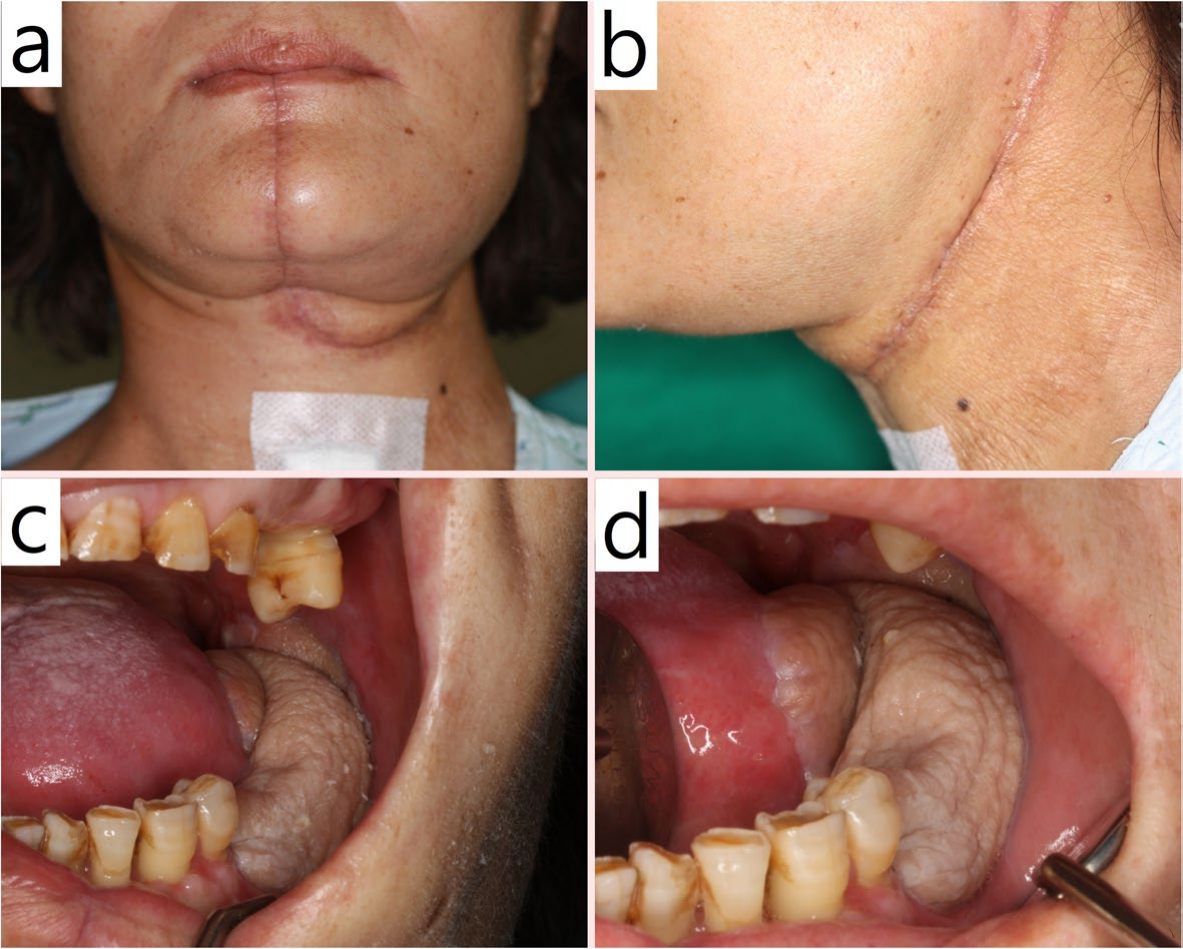
Postoperative photo after 1 month on patient No. 6 of group with tracheostomy. **a**, **b** Tracheostomy healed site. **c**, **d** Intraoral wound state with forearm free flap setting

Although reviewing existing studies might suggest that tracheostomy should generally be performed as infrequently as possible, both this study and others have found numerous instances where tracheostomies effectively resolved emergency situations. Mishra S’s study [8], which suggested that nasal intubation could be a sufficiently safe alternative, reported that out of 260 patients, 3 required emergency tracheostomies due to upper airway obstruction symptoms after extubation. Similarly, in this study, patient number 7 in the tracheostomy group, who was initially maintaining their airway with nasal intubation alone, underwent an emergency tracheostomy on the eighth day post-surgery due to respiratory suppression even though nasal intubation was being maintained. Another patient in the tracheostomy group (patient number 6) faced an emergency situation due to sudden bleeding at the surgical site, which posed a high risk of airway obstruction, but was safely managed because the tracheostomy had been performed preoperatively. This patient had an oral squamous cell carcinoma extending to the base of the tongue and adjacent mandibular alveolar bone, and underwent reconstruction using a radial free flap after tongue cancer resection. On the second day post-surgery, sudden delayed bleeding caused by anticoagulants used to improve the free flap’s blood flow led to a hematoma at the base of the tongue, causing airway obstruction. However, the preoperatively performed elective tracheostomy secured the airway, allowing the patient to easily undergo general anesthesia and recover without life-threatening complications, eventually being discharged in normal condition. The patient has been regularly followed up for the next 10 years and has remained cancer-free. This patient’s Cameron score was 7 points, well above the threshold of 5 points, indicating a situation where tracheostomy might be considered necessary. This case highlights the importance of elective tracheostomy in appropriate indications before surgery (Figs. 1 and 2).

In this study, due to the relatively small sample size, specific evaluations such as sensitivity and specificity were not conducted. However, according to the study reported by Benatar-Haserfaty et al. [15], the Cameron tracheostomy scoring system demonstrated a diagnostic sensitivity value of 0.7 and a diagnostic specificity value of 0.9 within a 95% confidence interval. This indicates that the Cameron assessment method is significant for the objective determination of performing tracheostomies.

Despite the utility of the Cameron scoring system, there are limitations and areas for improvement in its structure. According to the scoring criteria, a single point is assigned for any mandibulectomy, regardless of whether it is marginal or segmental. This fails to account for the clear differences in postoperative swelling and discomfort between the two types, yet both are scored as a single point. Similarly, bilateral neck dissection is assigned 3 points, while unilateral neck dissection is scored the same as no neck dissection at 0 points, rather than being assigned 1 or 2 points. This issue is also present in another tracheostomy evaluation system, the TRACHY scoring system [6], where bilateral surgery scores 3 points and unilateral surgery 0 points. In research by Janik [16] that compared tracheostomy scoring systems, both the Cameron and TRACHY systems recognized bilateral neck dissection as a risk factor, but it was suggested that bilateral neck dissection itself is not a reliable predictor for elective tracheostomy. Although bilateral neck dissection may increase the risk of postoperative swelling and airway obstruction compared to unilateral procedure, the wide point gap between 0 and 3 points indicates a limitation in providing a more detailed prediction. Additionally, as the T stage of the tumor progresses and the tumor size increases, the extent of surgery expands, potentially leading to greater difficulties in airway management. The Cameron system does not include factors related to tumor size and stage, which could hinder a more accurate preoperative airway assessment. On the other hand, the scoring items related to the surgical site are the most detailed in the Cameron system compared to other scoring systems. The high scores assigned for surgeries in the floor of the mouth and oropharynx regions, areas that can significantly impact airway maintenance, reflect the higher risk associated with surgeries in these areas. This detailed assessment is likely why the Cameron system showed the highest concordance in comparison with other scoring systems in this study.

The TRACHY score system includes additional factors such as the size and anatomical location of the primary lesion, overall health status like the ASA grade, and whether the patient had received radiation therapy. Additionally, there are other scoring systems, such as the one developed by Leiser [14], which differentiates glossectomy and mandibulectomy into three categories based on extent and assigns scores according to T staging. Furthermore, the scoring system by Gupta et al. [7], known as CASST (Clinical Assessment Scoring System for Tracheostomy), was also introduced. This system identifies major risk factors including radiation therapy, bilateral neck dissection, resection of more than half of the mandible, and large reconstructive flaps, and also classifies reoperation, age over 65, trismus, and abnormal findings on chest CT like COPD as minor risk factors.

By applying the TRACHY and CASST systems mentioned above to the cases in this study and comparing the results (Tables 4 and 5), we found that in the TRACHY system, 4 out of 10 patients in the tracheostomy group scored the threshold score (4 points) or higher, while none did in the non-tracheostomy group. In the CASST system, only 1 out of 10 patients in the tracheostomy group scored or exceeded the threshold score (7 points), and all 19 patients in both the tracheostomy and non-tracheostomy groups were below the threshold. In contrast, the Cameron system applied in this study showed that all 10 patients in the tracheostomy group met the threshold score of 5 or higher, accurately reflecting the actual performance of tracheostomies. This confirms the usefulness of the Cameron system. When applied preoperatively, it may lead to more tracheostomies, potentially reducing the likelihood of encountering emergency situations. However, as noted in the study by Schmutz et al. [11], there is a concern that the scoring system could result in unnecessary tracheostomies. Conversely, while applying other systems might reduce the likelihood of unnecessary tracheostomies, it could also increase the risk of being unprepared for emergency situations. Therefore, it is essential to consider multiple scoring systems while also being proactive about performing tracheostomies. Airway obstruction during major oral cancer surgery cannot be entirely predicted by scoring alone. Consequently, it is prudent for surgeons to perform tracheostomies in more patients when risks are anticipated, as supported by other literature.

Literature [16, 17] comparing these systems commonly concludes that none of the proposed scoring systems can directly predict postoperative hematoma and pharyngeal swelling. However, the benefits of tracheostomy include preventing pneumonia and reducing the risk of iatrogenic flap rupture during re-intubation processes, demonstrating other additional advantages.

Therefore, it is advisable to consider elective tracheostomy more actively before surgery, not only in cases where the Cameron scoring score is above the threshold, but also when complications such as bleeding or swelling in areas adjacent to the upper airway are anticipated postoperatively. Adopting this proactive approach in anticipation of emergency situations is by no means considered an excessive measure. Additionally, the presence of comorbidities investigated in this study did not significantly affect the performance of elective tracheostomy in either patient group.

This research did not apply the Cameron score system prospectively before surgery to directly influence decisions during cancer surgeries. Instead, the cases were analyzed retrospectively, postoperatively, which may not accurately reflect the role of the proposed scoring system. Despite grouping without prior knowledge of the Cameron score and performing a retrospective analysis based on cases where the decision to perform a tracheostomy had already been made, significant differences in Cameron scores between the two groups were observed. The actual performance of tracheostomy also matched perfectly with the Cameron scores for all 10 patients. This confirms the clinical usefulness of the Cameron scoring system.

Through retrospective examination of patient cases and a review of the literature, this study identified clinical factors that increase the necessity for elective tracheostomy and presented actual cases where preemptively performed tracheostomies during emergencies involving upper airway obstruction were resolved without significant issues. It also reviewed similar cases in the literature. Through these analyses, this study reconfirmed the usefulness of elective tracheostomy in surgeries for patients with severe oral cancer and is likely to increase awareness among surgeons.

## Conclusion

Appropriate airway management is crucial for the postoperative recovery and care of oral cancer patients. The advantages of preoperative elective tracheostomy include preventing damage to the surgical site and free flaps during re-intubation processes in emergencies such as respiratory failure due to severe bleeding from the surgical site, significant neck swelling, or vascular surgeries needed to maintain the viability of free flaps due to vascular thrombosis. Additionally, it offers benefits such as preventing postoperative complications like pneumonia. Furthermore, a retrospective study applying the Cameron scoring system, previously proposed as a predictor of postoperative respiratory failure, to 20 oral cancer surgery patients at our institution confirmed the usefulness and efficacy of both the scoring system and tracheostomy. In advanced oral cancer surgery, it is essential to actively consider the application of elective tracheostomy by using various scoring systems that predict postoperative respiratory failure and assess the risk of airway obstruction before surgery. Tracheostomy is considered to significantly aid in proper airway management during oral cancer surgery.

## Data Availability

All data generated or analyzed during this study are included in this published article.

## Abbreviations

ICU: Intensive care unit
ASA: American Society of Anesthesiologists
RFFF: Radial forearm free flap
RT: Radiation therapy
CT: Computed tomography
COPD: Chronic obstructive pulmonary disease
SOHND: Supraomohyoid neck dissection
SND: Selective neck dissection
FOM: Floor of mouth
FFF: Fibula free flap
mRND: Modified radical neck dissection
bND: Bilateral neck dissection
